# Unlocking beta cell health: The clinical potential of extracellular vesicles in type 1 diabetes

**DOI:** 10.1002/ctm2.70700

**Published:** 2026-05-18

**Authors:** Nanthini Jayabalan, Flavio Carrion, Kriti Joshi, Tony Huynh, Carlos Salomon

**Affiliations:** ^1^ Translational Extracellular Vesicles in Obstetrics and Gynae‐Oncology Group Frazer Institute Faculty of Health, Medicine and Behavioural Sciences The University of Queensland Brisbane Queensland Australia; ^2^ UQ Centre for Extracellular Vesicle Nanomedicine Faculty of Health, Medicine and Behavioural Sciences The University of Queensland Brisbane Queensland Australia; ^3^ Departamento de Investigación Postgrado y Educación Continua (DIPEC) Facultad de Ciencias de la Salud Universidad del Alba Santiago Chile; ^4^ Department of Endocrinology and Diabetes Queensland Children's Hospital South Brisbane Queensland Australia; ^5^ Department of Chemical Pathology Mater Pathology South Brisbane Queensland Australia; ^6^ Child Health Research Centre Faculty of Health Medicine, and Behavioural Sciences The University of Queensland South Brisbane Queensland Australia

**Keywords:** biomarkers, extracellular vesicles, immunomodulation, precision medicine, type 1 diabetes

## Abstract

**Background:**

Type 1 diabetes (T1D) is a lifelong autoimmune disease characterised by progressive immune‐mediated destruction of insulin‐producing beta (β)T1D‐cells, leading to permanent insulin dependence and increased risk of microvascular and macrovascular complications. Despite advances in autoantibody screening and immunotherapies, major clinical challenges persist in early detection, accurate disease staging, prediction of progression and monitoring of therapeutic response. Current biomarkers provide limited insight into real‐time β‐cell stress and immune activity, restricting opportunities for timely and personalised intervention.

**Rationale:**

Extracellular vesicles (EVs) are nano‐sized membrane‐bound particles released by virtually all cell types and carry proteins, lipids and nucleic acids reflective of their cellular origin and physiological state. Advances in EV isolation, multi‐omics profiling and bioinformatics now enable detailed characterisation of EV cargo from accessible biofluids such as blood and urine. These developments position EVs as a minimally invasive platform to interrogate β‐cell health, immune activation and systemic complications in T1D, while also offering a novel class of cell‐free immunomodulatory therapeutics.

**Content:**

This review synthesises current evidence on the role of EVs in T1D pathogenesis and clinical translation. We discuss how β‐cell‐ and immune cell‐derived EVs participate in antigen presentation, immune activation and inflammatory amplification, and how EV cargo signatures (proteins, miRNAs and other RNAs) reflect disease stage, progression and heterogeneity. We summarise emerging data on maternal, neonatal and urinary EVs as early‐life and complication‐associated biomarkers, and critically evaluate ongoing EV‐based clinical studies in T1D. Finally, we examine the therapeutic potential of stem cell‐derived and engineered EVs to modulate autoimmunity and preserve residual β‐cell function.

**Conclusion:**

EVs introduce a potentially clinically actionable layer of information linking cellular stress, immune dysregulation and tissue damage to measurable biomarkers and therapeutic opportunities in T1D. However, the majority of EV applications currently remain at the preclinical or early pilot‑study stage, with limited validation in large, longitudinal patient cohorts. Key challenges include biological heterogeneity, assay reproducibility and the need for standardised isolation, characterisation and regulatory frameworks. While rapid advances in EV technologies and early proof‑of‑concept clinical studies support their long‑term potential, substantial work is required before routine clinical implementation is feasible. For feasible clinical translation of EV‐based applications, alignment with regulatory frameworks must be considered early to ensure analytical validity, standardisation and compliance with clinical and diagnostic approval pathways, as well as to address safety, efficacy and manufacturing requirements for EV‐based therapeutics.

**Key points:**

- EVs provide a minimally invasive window into β‐cell stress, immune activation and disease progression in T1D.
- EV‐associated proteins and RNAs reflect disease stage, heterogeneity and response to immunotherapy beyond traditional biomarkers.
- Circulating and urinary EVs show promise for early detection of T1D complications before clinical manifestation.
- Stem cell‐derived and engineered EVs represent emerging cell‐free immunomodulatory and regenerative therapies.
- Standardisation, longitudinal validation, EV‐focused clinical trial design and early alignment with regulatory frameworks are essential for clinical translation, ensuring robust validation for diagnostics and addressing safety, efficacy and manufacturing requirements for EV‐based therapeutics.

## Graphical abstract

Extracellular vesicles serve as minimally invasive biomarkers of β‑cell stress and immune dysregulation in T1D. This review highlights the potentials of EVs as a biomarker to improve early detection and disease staging in type 1 diabetes.

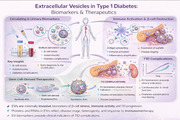

## 1 INTRODUCTION

T1D is the most common chronic childhood disease and represents a substantial and growing global health challenge. According to the International Diabetes Federation, approximately 589 million adults are currently living with diabetes, a number projected to increase by 46% by 2050. As of 2024, an estimated 9.2 million people worldwide are living with T1D, including 1.8 million children and adolescents under the age of 20 years.^1^

T1D is an autoimmune condition characterised by the presence of islet autoantibodies as a hallmark of immune dysregulation, and the progressive destruction of insulin‐producing β‐cells within the pancreatic islets mediated primarily by autoreactive T lymphocytes. These pathogenic processes ultimately culminate in the development of clinical T1D.^2^ This chronic condition necessitates lifelong insulin replacement therapy, presenting an ongoing burden that, coupled with the continuously increasing global prevalence, underscores the critical need for screening and monitoring of individuals at risk for T1D development.

There has been a paradigm shift in the definition of T1D with a focus on early diagnosis and detection. T1D can be divided into different stages based on the presence of multiple islet autoantibodies and the level of glycaemic dysregulation Stage 1 with normoglycaemia, Stage 2 with dysglycaemia and Stage 3 with biochemical diabetes with or without symptoms.^3^, ^4^ Metabolic testing underpins this classification. The oral glucose tolerance test remains the gold standard and most sensitive method for detecting early dysglycaemia in Stage 2 but its utility is limited by challenges in its performance in young children, a lack of reproducibility due to significant day‐to‐day variability and the lack of correlation with glucose handling related to normal daily dietary consumption.

Glycated haemoglobin (HbA1c) offers good specificity but less sensitivity for the diagnosis of early stage T1D^5^ as well as lacking the ability to capture day‐to‐day variability in glycaemic excursions characteristic of the early stages of T1D. Continuous glucose monitoring (CGM) can reveal subtle glucose excursions that predict progression, but CGM metrics requires further studies to validate its utility in T1D staging. There remain gaps in the methods for staging T1D and predicting the rate of progression through early stages of T1D to clinical diabetes.

Islet autoantibody screening and staging of T1D in first‐degree relatives of patients with T1D is increasingly being adopted into clinical practice due to ongoing immunotherapy trials,^6^ and since the approval by the United States Food and Drug Administration of teplizumab for individuals with Stage 2 T1D to slow progression to clinical T1D.^7^ However, assessment and prediction of treatment response to immunotherapy currently relies on various iterations of C‐peptide measurements as a marker of residual β‐cell function.^8^, ^9^ The utility of this approach is limited as it is an indirect marker of any potential changes in the pathophysiological process, requiring repeated measures over the course of intervention. C‐peptide also does not reflect endogenous β‐cell stress. It is a marker that does not account for the heterogeneity of T1D pathophysiology,^10^ which may require more specific and targeted therapies for different individuals.

**FIGURE 1:**
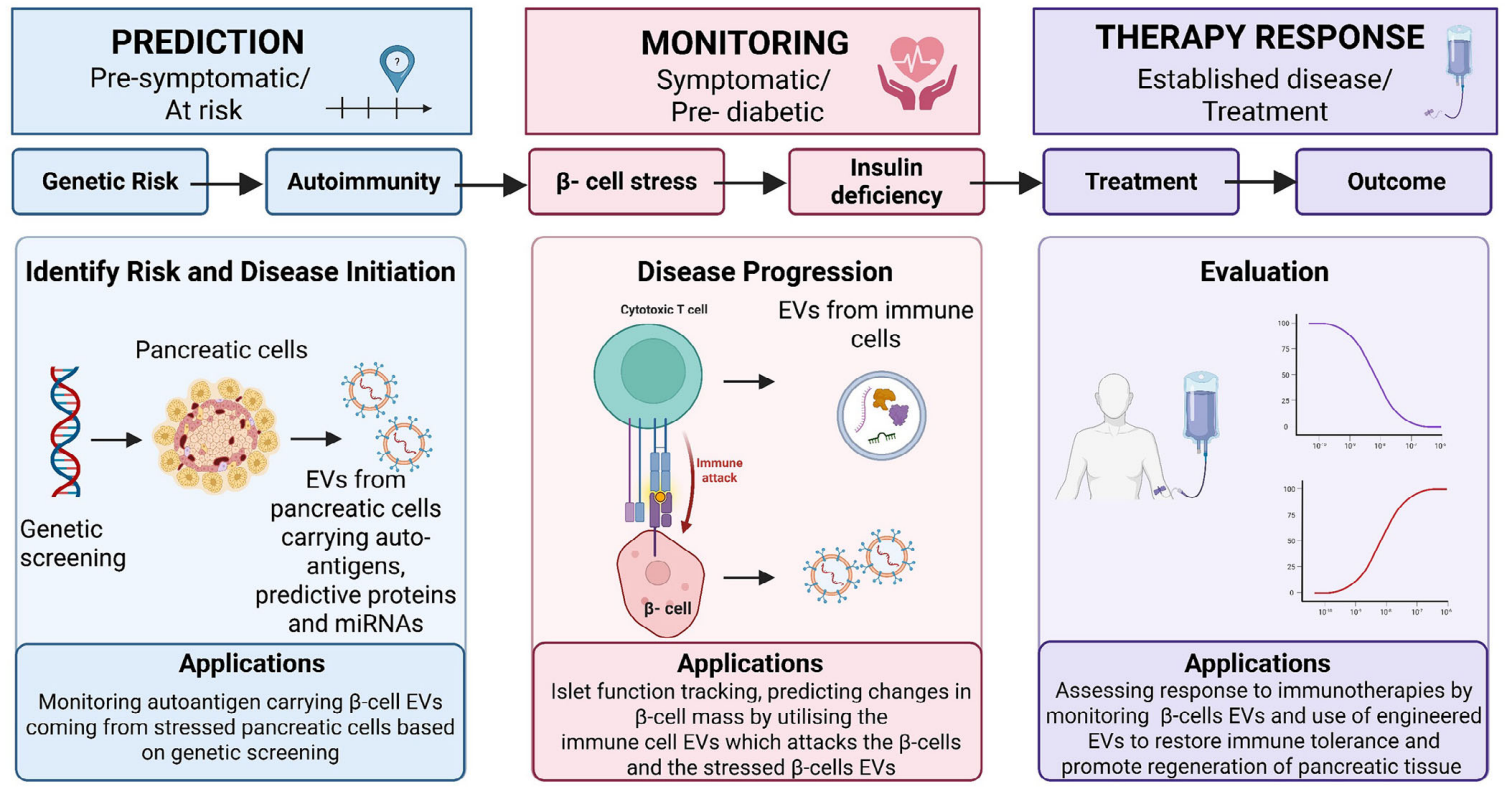
The role of EVs across the T1D disease continuum.

In recent years, EVs have emerged as highly promising biomarker candidates. Their appeal stems from their ability to reflect the physiological and pathological state of their parent cells, their remarkable stability and the ease with which they can be isolated from various biofluids.^11^, ^12^, ^13^ This review paper will explore the promising role of EVs as dynamic biomarkers for T1D, monitoring and progress of T1D as well as the treatment outcome (Figure 1). This review is directed towards clinician‐scientists, endocrinologists and translational researchers and examines the potential role of EV–based biomarkers within secondary and tertiary care pathways, particularly in diabetes and endocrinology clinics, rather than as tools for population‐wide primary screening.

This figure illustrates the dynamic roles of EVs throughout the progression of T1D, from pre‑symptomatic risk to established disease and therapeutic intervention. In the prediction phase, EVs derived from pancreatic and immune cells carry genetic material, autoimmunity‑related biomarkers and regulatory microRNAs that may help identify individuals at risk and inform early genetic or immune‑profiling strategies. During disease monitoring, shifts in EV cargo reflect β‑cell stress, immune activation and metabolic dysregulation. These EV signatures allow tracking of disease progression from pre‑diabetic to symptomatic stages and provide insight into β‑cell loss. In the therapy response stage, EVs can serve as minimally invasive indicators of patient responsiveness to immunotherapies or emerging engineered EV‑based treatments. The ability of EVs to mimic immune activity and β‑cell health positions them as promising biomarkers for evaluating therapeutic efficacy and supporting personalised T1D management. Applications across all three stages highlight EVs as a versatile platform for early detection, monitoring and treatment response assessment in T1D.

EVs are heterogenous, phospholipid bilayer membrane‐derived vesicles playing key roles in cell‐to‐cell communication and conveying molecular signals to cells at proximal as well as distal locations.^14^, ^15^ The EVs are classified into multiple subtypes based on their origin and size. The major subpopulations of EVs are including exosomes, ectosomes, oncosomes and apoptotic bodies.^16^ Exosomes are typically 30–200 nm in size and originate from the multivesicular endosomes whereas the medium EVs, ectosomes, namely, microparticles (MP) and microvesicles (MVs), are >200 nm in size and originate from the plasma membrane. The apoptotic bodies are released from cells undergoing programmed cell death and ≥1000 nm in size.^16^ EVs are enriched with a wide range of molecular cargo, including proteins, lipids, messenger RNAs (mRNAs) and microRNAs (miRNAs), reflecting the composition of their parent cells and the microenvironment of their origin.^17^, ^18^ This unique characteristic underpins their role in intercellular communication and diverse biological processes, making EVs an attractive and informative biomarker platform for studying pathological conditions.

## 2 EVs IN T1D: A NON‐INVASIVE WINDOW TO Β‐CELL HEALTH

The early and accurate diagnosis of T1D is paramount for effective intervention and improved patient outcomes. Traditional diagnostic methods to detect evidence of islet autoimmunity primarily rely on islet autoantibody panels, C‐peptide levels and glycaemic indicators, often preceding clinical diagnosis by years. During this period, a substantial portion of β‐cell mass can be irrevocably lost, diminishing the window for effective disease‐modifying or preventative interventions. The pursuit of minimally invasive, highly sensitive and specific biomarkers is a critical frontier in modern medicine, particularly for complex autoimmune diseases like T1D.

As dynamic carriers of a diverse molecular cargo providing a health snapshot of their parent cell, EVs have emerged as highly promising biomarkers. EVs are well recognised as early biomarkers for various autoimmune diseases, such as rheumatoid arthritis, systemic lupus erythematosus and multiple sclerosis where changes observed across EVs number, contents including protein, mRNA and miRNA.^19^, ^20^, ^21^ In this context, a comparative study reported a twofold increase in circulating EV levels in individuals with T1D compared with healthy controls. However, the study did not discriminate between early and late disease stages.^22^ This pattern is echoed in another study that noted a 2.5‑fold elevation in circulating EV levels among diabetic subjects, although disease staging was not specified.^23^ Consistent with these findings, Sabatier et al.^24^ also reported elevated circulating MP levels in individuals with T1D. Interestingly, the study by Garcia‐Contreras et al.^25^ found no significant differences in circulating exosomes abundance between healthy individuals and those with T1D and the disease stage of the participants was not specified. In this regard, a very few studies have attempted to examine circulating EV levels and the compositions in relation to various stages of T1D. This highlights a substantial gap in current knowledge and underscores the urgent need for systematic, stage‐specific research to determine how EV dynamics evolve across the continuum of T1D development.

On the other hand, numerous studies have highlighted significant alterations in the molecular cargo of EVs in T1D. EVs released by pancreatic β cells^26^, ^27^, ^28^ carry molecular signatures directly reflective of their origin and physiological state. This offers an unprecedented level of granularity compared with traditional systemic biomarkers. Exosomes^28^ and total EVs^29^ derived from β‐cells, for instance, have been shown to carry autoantigens such as glutamic acid decarboxylase 65 (GAD65) and zinc transporter 8 (ZnT8). The presence of these antigens within EVs signals an ongoing immune attack on β‐cells. miRNAs are short, ∼22 bp long non‐coding RNA molecules and involved in the regulation of post‐transcriptional gene expression by inhibiting mRNA transcription or by repressing protein translation.^30^ The release of cellular miRNAs into extracellular space is usually associated with carriers, such as lipoproteins,^31^ ribonucleoproteins^32^ or EVs.^33^ Among these, miRNAs released into extracellular space via the exosomes comprise the largest fraction of circulating miRNAs.^34^

In this context, exposure of β‐cells to stressors, such as inflammatory cytokines, has been observed to increase the expression of miR‐21‐5p in exosomes.^35^ miR‐21‐5p, a miRNA associated with apoptosis,^36^ has specifically been shown to increase in circulating exosomes prior to T1D onset and in children with new‐onset T1D.^35^ Hence, the unique molecular signatures of EVs, accurately reflecting the physiological and pathological state of β‐cells, present a compelling opportunity for their use as non‐invasive biomarkers for the early detection of T1D.

## 3 EVs IN IMMUNE ACTIVATION AND ANTIGEN PRESENTATION IN T1D

The crosstalk between pancreatic β‐cells and T‐cells is the central axis of T1D pathogenesis. The β‐cells are not just the victim of the immune system but act as active participant that communicates to T‐cells, leading to accelerating their destruction. The endoplasmic reticulum (ER) stress in the β‐cells as well as the expression of chemoattractants up‐regulate the expression of HLA Class I^37^ with the presentation of neoantigens^38^, ^39^ which attracts the T‐cells and the binding of T‐cells to these neoantigens on the β‐cells triggers apoptosis.^40^, ^41^

Besides the direct communication, EVs facilitate the communication between β‐cells and T‐cells. For instance, human islet‐derived total EVs have been shown to directly activate both T‐ and B‐cells from T1D patients, leading to an increase in GAD65 autoantibody production, thereby directly implicating them in the specific immune response to autoantigens.^42^ Furthermore, conditions such as ER stress enhance the secretion of EVs from β‐cells. These stress‐induced EVs carry crucial immunomodulatory chaperones and autoantigens, which are subsequently presented to antigen‐presenting cells, effectively initiating and amplifying an immune response.^28^ Similarly, β‐cells exposed to pro‐inflammatory cytokines produce CXCL‐10 enriched exosomes, which bind to CXCR3 receptors on autoreactive T‐cells. This binding promotes the chemotaxis of T‐cells towards β‐cells, thereby perpetuating an inflammatory feedback loop and contributing to β‐cell death.^43^ Collectively, these findings underscore EVs as indispensable messengers, fundamentally facilitating the destructive crosstalk between pancreatic islet cells and the immune system in T1D. This intricate EV‐mediated dialogue positions them as central players in T1D disease pathogenesis and highlights their potential as a biomarker for early disease diagnosis.

## 4 EVs AS IMMUNOMODULATOR IN T1D

EVs are increasingly recognised as potent immunomodulators in T1D, shaping the immune response towards the β‐cells and influencing disease progression. While EVs from dying β‐cells are demonstrated to contribute to autoimmunity, recent studies have also highlighted a protective role for EVs originating from the β‐cells. The dendritic cells pulsed with pancreatic β‐cells apoptotic bodies (NIT‐1) induced antigen‐specific tolerance in NOD mice, effectively preventing T1D.^44^ By promoting the peripheral tolerance pathways via efferocytosis, the therapy reduces insulitis and maintains normoglycaemia in the NOD mouse model, offering a potential cellular therapy for autoimmune diseases. Programmed death‐1 (PD‐1) is an inhibitory receptor found on activated immune cells, which inhibits immune responses upon binding to its ligand, programmed death‐ligand 1 (PD‐L1).^45^ Previous studies have shown the expression of PD‐L1 in β‐cells as an act to limit destruction by immune cells.^46^, ^47^ A very recent study showed the expression of PD‐L1 on the surface of exosomes released by the β‐cells in those who are autoantibody positive as well as in patients with recent onset T1D.^48^ Crucially, these circulating exosomal‐PD‐L1 levels were observed to correlate with C‐peptide in these individuals. Besides being protective, the expression levels on PD‐L1 on the surface of EVs can be used as a biomarker to track disease progression and heterogeneity.

## 5 PROTEOMIC AND TRANSCRIPTOMIC PROFILING OF EVs IN T1D

By encapsulating and displaying a diverse array of proteins and various RNA species (including mRNAs, miRNAs and long non‐coding RNAs) that recapitulate the phenotype of their originating cells, circulating EVs offer a non‐invasive window into disease mechanisms. Proteomic profiling of EVs allows for the identification of differentially abundant proteins, which can indicate β‐cell stress or immune activation and even help distinguish T1D disease stages or endotypes. To further explore the potential of exosomes as biomarkers in T1D, a discovery study conducted comprehensive proteomic and phosphoproteomic analyses of circulating exosomes, identifying 181 differentially abundant proteins and eight phosphoproteins in T1D subjects relative to controls.^49^ These distinctive exosomes signatures were significantly associated with critical cellular processes, including immune activation and MHC class II signalling. Importantly, specific protein modules identified through weighted gene co‐expression network analysis demonstrated significant correlations with characteristic clinical features, such as pancreas size, underscoring their relevance to T1D pathophysiology.

Besides the earlier mentioned miR‐21‐5p, the transcriptomics of exosomes demonstrated the enrichment of miR‐375, a β‐cell enriched miRNA in the circulation of T1D patients.^50^

Giri et al.^51^ demonstrated that β‐cells exposed to stressors release various EV subtypes enriched with immune‑stimulatory miRNA sequences, which may play a role in activating immune responses and contributing to the pathogenesis of T1D. Other miRNAs enriched in EVs obtained from patients with T1D include miR‑29, miR‑122‑5p, miR‑192‑5p, miR‑455‑5p and miR‑185‑5p, which are primarily involved in the regulation of immune activation.^50^, ^51^, ^52^, ^53^

mRNAs are single stranded molecules that mediate the transfer of genetic information from nuclear DNA to the cytoplasm to direct protein synthesis.^54^ The EVs exhibit a selective enrichment of mRNA, a feature that can be exploited for diagnostic purposes and for monitoring disease progression.^55^ For example, Wilms’ Tumour 1 (WT‐1), a transcription factor selectively expressed in podocytes that are essential for maintaining the glomerular filtration barrier,^56^ shows elevated mRNA levels in circulating exosomes from T1D patients with diabetic nephropathy. This increase occurs concomitantly with reduced expression of angiotensin converting enzyme mRNA.^57^ The comprehensive exosomes‐associated mRNA sequencing has further revealed the differential regulation of 66 up‐regulated and 46 down‐regulated transcripts. Among these, four mitochondrial mRNAs – MT ND2, MT ND5, MT ND3 and MTRNR2L12 – have been implicated in T1D related pathophysiological processes, including the modulation of β‐cell susceptibility to T‐cell‐mediated cytotoxicity.^58^ Collectively, these findings underscore the potential of EV mRNA profiling to elucidate disease mechanisms and to inform the development of novel, non‐invasive biomarkers for T1D.

## 6 MATERNAL AND NEONATAL EV SIGNATURES IN T1D

Maternal and neonatal EV signatures are increasingly recognised as critical determinants of early‐life immune programming and T1D susceptibility, with substantial implications for biomarker discovery. Maternal EVs may act as vehicles for immune‐modulatory signals that shape neonatal immune development during key developmental windows. Recent studies of breast milk‐derived exosomes have identified significantly dysregulated miRNA cargo in mothers with T1D compared with healthy controls.^59^ In particular, enrichment of miR‐4497 and miR‐3178 has been shown to enhance TNF production in human macrophages, suggesting a pro‐inflammatory imprinting mechanism mediated by maternal exosomes. These findings implicate breast milk EV‐associated miRNAs as functional mediators of early immune education and position them as non‐invasive biomarkers reflecting maternal autoimmune status and potential disease risk transmission. In a similar study, profiling of exosomes from the circulation of lactating mothers has revealed increased abundance of miRNAs including miR‐146a‐5p, miR‐26a‐5p, miR‐24‐3p and miR‐30d‐5p, which are  implicated in immune regulation and pancreatic β cell function.^60^ Although the direct causal relationship between altered maternal systemic EV profiles and offspring T1D risk remains to be established, these data collectively point to a perturbed maternal immunological milieu that may prime genetically susceptible infants towards early autoimmune trajectories. Taken together, these studies underscore the potential of circulating and breast milk‐derived EV miRNAs as dynamic, accessible biomarkers, offering mechanistic insight into maternal influences on T1D pathogenesis and providing a foundation for early risk stratification strategies.

## 7 EVs IN T1D COMPLICATIONS AND COMORBIDITIES

The systemic nature of diabetes means that sustained hyperglycaemia and chronic inflammation contribute to damage across multiple organ systems. T1D is frequently associated with increased risk of various diseases such as nephropathy,^61^ retinopathy^62^ and other autoimmune diseases.^63^, ^64^ Among the traditional biomarkers used for screening comorbidities in T1D are microalbuminuria for nephropathy,^65^ fundus examination for retinopathy^66^ and blood pressure and lipid profile monitoring for cardiovascular dysfunction.^67^ However, current screening strategies are primarily designed to detect clinically evident disease and lack the sensitivity to identify subclinical changes, which is crucial for preventing disease progression.

This strengthens the need for more sensitive and specific biomarkers that can detect the very earliest signs of complications in T1D, enabling proactive management rather than reactive treatment of established disease. Urinary EVs have emerged as particularly promising non‐invasive biomarkers for the early detection and monitoring of diabetic nephropathy. EVs shed from various kidney cell types into the urine carry a molecular cargo that reflects the real‐time health and stress of the renal system, often preceding changes in traditional markers like albuminuria. For instance, Lytvyn et al.^68^ showed a significant increase the levels of urinary podocytes‐derived MPs compared with healthy controls, offers a promising tool for identifying T1D patients at the risk for developing kidney complications.

Neutrophil gelatinase‐associated lipocalin (NGAL) is a sensitive marker of renal dysfunction and kidney injury.^69^ This marker of tubular injury has been detected in urinary EVs and has been shown to be a more frequent and higher indicator of early kidney damage in paediatric patients with T1D compared with non‐EV‐associated NGAL.^70^ Urinary exosomes‐associated NGAL correlates with T1D duration and HbA1c levels, independent of the albumin to creatinine ratio. In T1D, damage to podocytes leads to the release of the previous described WT‐1 protein into the urine.^56^ In relation to this, the analysis of exosomes released by the injured podocyte injury showed elevated presence of WT‐1 protein.^71^

Similarly, the increased expression of cystatin B and altered protease profiles in urinary exosomes were reported which may serve as biomarkers of kidney damage in T1DM.^72^ Besides EVs‐derived proteins, specific mRNAs and miRNAs within urinary EVs show significant potential. MiR‐145, for example, has been observed to be up‐regulated in urinary exosomes from diabetic patients with micro‐albuminuria, and its increase correlates with proteinuria development, suggesting a role as a biomarker of damage progression.^73^

The early identification of such subtle renal dysfunction through urinary EV analysis is especially critical for paediatric T1D populations, where proactive management based on these biomarkers could significantly alter the trajectory of disease, potentially delaying or preventing the onset of advanced kidney disease and its associated morbidity. In patients with T1D, circulating levels of endothelial‐ and platelet‐derived MPs are frequently elevated, reflecting ongoing cellular activation, stress and damage within the vascular system.^74^ Importantly, these EVs extend beyond their role as indicators of injury and actively contribute to disease pathogenesis by disseminating pro‑inflammatory and pro‑thrombotic signals. For instance, High Mobility Group Box 1 (HMGB1), a potent pro‐inflammatory molecule that plays a central role in the pathogenesis of T1D. It serves both as a biomarker of disease activity and a driver of pancreatic islet destruction. Upon release from damaged pancreatic cells, HMGB1 amplifies autoimmune responses by impairing regulatory T‐cell function and activating inflammatory signalling pathways through receptors such as TLR4 and RAGE.^75^ Elevated levels of this alarmin have been detected in endothelial and platelet MVs from individuals with T1D, particularly in those with microvascular complications.^74^ The transfer of such molecules via EVs can directly impact recipient cells, exacerbating inflammation, promoting oxidative stress and impairing endothelial function, thus contributing to the initiation and progression of endothelial dysfunction. Therefore, profiling these specific EV populations and their pro‐inflammatory cargo offers a valuable avenue for identifying T1D patients at heightened risk of cardiovascular morbidity.

## 8 EVs IN T1D DIAGNOSTICS AND THERAPEUTICS‐BASED CLINICAL TRIALS

Table 1 summarises registered clinical studies investigating EVs in the context of T1D, providing an overview of the current translational landscape and its limitations. Most trials focus on circulating blood‐derived EVs within observational or biomarker‐discovery frameworks, reflecting the early stage of clinical EV research in T1D. These studies typically examine metabolic stress, autoimmune features or early disease detection and are often embedded within broader physiological or interventional protocols (e.g. mixed meal tolerance tests or lipid‐lowering therapy), rather than being designed as EV‐centric clinical trials. Consistent with the exploratory nature of the field, many outcomes remain unpublished or studies are ongoing.

**TABLE 1:** List of EV‐based clinical trials in T1D.

Study #	Study title	Study description	Study type	Study design/intervention	Primary measures
NCT02138331	Effect of microvesicles and exosomes therapy on β‐cell mass in T1D	This study hypothesised that intravenous infusion of cell free umbilical cord‐blood‐derived MSC microvesicles may reduce the inflammatory state and hence improve the β‐cell mass as well as the glycaemic control of the patients of T1D.	Interventional	Two‐step intravenous infusion of cell‐free vesicles derived from umbilical cord‐blood mesenchymal stem cells (CB‐MSCs) First dose at Day 0: purified exosomes at a dose of 1.22–1.51 × 10^6^/kg/IV Second dose at Day 7: purified microvesicles at a dose of 1.22–1.51 × 10^6^/kg/IV	To measure the change in the amount of external insulin a patient requires; a successful result would be a significant reduction in the units of insulin needed per day while maintaining stable blood sugar (RBS between 120 and 160 mg/dL). The secondary measure of this study is the pancreatic β‐cells mass which was assessed by levels of C‐peptide. Since C‐peptide is created in a 1:1 ratio with natural insulin, higher levels indicate that the patient's own β cells are surviving or regenerating.
NCT04164966 (HONEY study)	Development of novel biomarkers for the early diagnosis of T1D	The purpose of this study is to measure the levels of certain substances (biomarkers) in the body that may indicate the triggers of T1D, to find a better way to diagnose the disease, as well as to follow its progression.	Observational	Isolation of β‐cell specific exosomes from the blood to analyse their protein and genetic cargo	To measure the concentration of circulating β‐cells specific exosomes in children with newly diagnosed T1D compared with healthy children; if successful, this would provide a ‘liquid biopsy’ of the pancreas without needing a physical tissue sample.
NCT06832215	Urine extracellular vesicles: non‐invasive biomarkers of Β‐cell function and novel therapeutic agents in diabetes (EvsBioDiabetes)	This project proposes the development of a non‐invasive biomarker of preclinical T1D, based on miRNA characterisation in urine, allowing a timely identification of children that can benefit from preventive therapies and, in the future, to cure T1D.	Observational	Blood and urine were collected at a single time point from individuals with T1D, their genetically related groups such as siblings and healthy control for the isolation of EVs.	To characterise the urine and blood EVs miRNA signature to identify a biomarker signature that can detect T1D in the pre‐clinical stage (Stage 1 or 2)
NCT07480161	Mesenchymal stem cell and exosome therapy for diabetic erectile dysfunction	This project aims to evaluate the effectiveness and safety of intracavernosal injections of umbilical cord (UC)‐derived mesenchymal stem cells (MSCs) and umbilical cord‐derived MSC‐derived exosomes in men with diabetic erectile dysfunction (ED) who have not responded adequately to conventional medical treatments.	Interventional	Participants randomised into 3 groups to receive a single treatment via intracavernosal injection. A: 5 × 10^6^ UC‐derived MSCs B: 75 µg MSC‐derived exosomes C: Placebo (saline)	The primary outcome measure is the change in erectile function assessed by the International Index of Erectile Function‐5 (IIEF‐5) and determine if the intracavernosal administration of MSCs or MSC‐derived exosomes improves erectile function.
NCT03392441	Insulin deprivation on brain structure and function in humans with	To determine the effects of transient insulin deprivation on brain structure, blood flow, mitochondrial function, cognitive function and circulating exosomes and metabolites in T1Dpatients	Interventional	T1D patients requiring insulin will have insulin stopped for a short period of 4–6 h.	Changes in structural MRI was taken at baseline and at 6 h post insulin deprivation. The specifics on circulating exosomes were not reported.
NCT07270172	The MICRON study—a steno 1 substudy (MICRON)	The goal of this observational study is to compare cardiac and renal oxygen consumption among subjects with T1D treated with either multifactorial intervention or according to the current standard care.	Observational	Participants from the main Steno 1 study will have specialised scans to measure heart and kidney oxygen use, kidney function tests, blood and urine samples will be collected.	The primary measure of this study is to see if the intensive, multifactorial treatment reduces the ‘workload’ on vital organs compared with standard care. At the 0, 6 and 24‐month visits, blood and urine samples will be collected to isolate EV to perform proteomics and to correlate the PET data with EV protein signature.
NCT03106246	Circulating extracellular vesicles released by human islets of Langerhans	To detect and characterise β‐cells derived EV in the circulation of new onset T1D, established T1D and T2D patients, patients who have received islet transplant and healthy controls.	Observational	Blood samples were collected at defined intervals to determine β‐cell specific EV and determine the utility of this biomarker as a measure of β‐cell stress or injury.	Based on well‐known EV markers, subject plasma samples will be characterised to determine whether these EVs are detectable using small particle flow cytometry and if these EVs contain islet‐specific antigens as GAD65 and ZnT8 and correlate these EVs to patient parameters like age, duration of established diabetes and levels of autoantibodies.
NCT03971955	Characterisation of adult onset autoimmune diabetes (CIAO)	The purpose of the study is to identify new biomarkers of adult onset autoimmune diabetes (AOnAD).	Observational	This study compared and characterised the clinical, metabolic and immunological features across individuals diagnosed with AOnAD, T1D, T2D and healthy controls.	The primary measure of this study is the changes in the levels of specific miRNAs isolated from circulating exosomes across the study groups.
NCT01497912	Treatment effects of atorvastatin on haemostasis and skin microcirculation in patients with type 1 diabetes	The aim of this study was to investigated the effects of intensive LDL‐cholesterol‐lowering therapy with atorvastatin on fibrin network permeability (primary variable) and other aspects of haemostasis in patients with T1D and dyslipidaemia.	Interventional	Twenty (20) adults (aged 30–70 years) with type 1 diabetes and dyslipidaemia were randomised into two groups. A: Participants took 80 mg Atorvastatin (a high dose) once daily for 8 weeks. Washout Period: A 2‐month break where no study medication was taken to ensure the drug cleared the system. Phase B: Participants took a matching placebo once daily for 8 weeks.	The primary outcome was fibrin network permeability and thrombin generation. Additionally, the concentration of platelet and endothelial microparticles were measured to investigate the effects on the treatment on the circulating vesicles.
NCT00934336	Importance in type 1 diabetes patients of an optimized control of post‐prandial glycaemia on oxidant stress prevention (ITOPOS)	To investigate if the timing of insulin injections and the resulting control of blood sugar spikes after meals could reduce oxidative stress that damages blood vessels in people with T1D	Interventional	To achieve pre‐prandial phase) versus post‐prandial phase and measure the biological fallout in T1D and healthy control; Pre‐prandial injection: Injecting an ultra‐fast‐acting insulin analogue before meals Post‐prandial: Injecting the same insulin after meals	The primary measure of this study was to measure the lipid peroxidation in urine sample. The secondary measure was to study the platelet and endothelial microparticles alongside studying the antioxidant measures.

Only a small number of trials extend beyond biomarker discovery towards therapeutic or non‐invasive applications. These include studies evaluating mesenchymal stem cell (MSC)‐derived EVs for β‐cell preservation (NCT02138331) and urine‐derived EVs (NCT06832215) as surrogates of β‐cell function. Collectively, the studies summarised in Table 1 focusing on T1D demonstrate growing clinical interest in EVs while underscoring the need for rigorously designed, EV‐focused trials with harmonised methodologies and clearly defined endpoints to enable clinical integration in T1D. The compiled clinical trials in Table 1 were searched in NCBI, ClinicalTrials.gov website using the keywords: T1D, diabetes, EVs, exosomes, MPs and MVs.

A substantial proportion of ongoing clinical research is centred on the diagnostic potential of EVs. For example, the study *Circulating Extracellular Vesicles Released by Human Islets of Langerhans* (NCT03106246) aims to characterise blood‐derived EVs associated with islet health and function, providing foundational insights into EV signatures linked to pancreatic islets. Similarly, *Characterisation of Adult‐Onset Autoimmune Diabetes* (NCT03971955) and *Development of Novel Biomarkers for the Early Diagnosis of Type 1 Diabetes* (NCT04164966) are ongoing studies using blood‐derived EVs to identify protein, lipid or nucleic acid cargo associated with β‐cell autoimmunity and disease progression, potentially complementing established diagnostic tools such as autoantibody testing. In parallel, a Mayo Clinic study (CLS‐2052106) is examining EV cargo dynamics during T1D progression, with a particular focus on CXCL10‐enriched β‐cell‐derived EVs in response to diabetogenic inflammatory cues. Expanding non‐invasive approaches further, one trial is investigating urine‐derived EVs as biomarkers of β‐cell function and as potential therapeutic agents (NCT06832215), offering a highly accessible strategy for disease monitoring.

The therapeutic exploration of EVs in T1D is largely driven by their capacity to transfer bioactive cargo and modulate immune and regenerative pathways in a cell‐free manner. Given the early stage of EV‐based therapeutics, current efforts predominantly focus on stem cell‐derived EVs, particularly those from MSCs, which are known to exert anti‐inflammatory, immunomodulatory and regenerative effects while avoiding the risks associated with cell transplantation.^76^, ^77^ Notably, the clinical trial registered under NCT02138331 is evaluating the impact of MSC‐derived EV therapy on β‐cell mass, representing an important step towards translating EV‐based interventions into T1D treatment strategies.

Collectively, registered clinical studies investigating EVs in T1D indicate that the field remains at an early translational stage. The majority of trials are observational and biomarker‑focused, aiming to characterise EV cargo as indicators of β‑cell stress, autoimmunity or disease progression. In contrast, therapeutic applications of EVs are rare, with only a small number of early‑phase, proof‑of‑concept studies exploring EV‑based interventions. Notably, there is a clear absence of large, longitudinal validation cohorts and no completed phase II or III clinical trials, underscoring major gaps in clinical readiness. Overall, the current clinical trial landscape highlights both the promise and the immaturity of EV research in T1D. Existing studies establish biological relevance but stop short of clinical validation, reflecting limitations in scale, duration and methodological harmonisation. Progress towards clinical integration will require a strategic shift from exploratory discovery to rigorous validation frameworks, including standardisation of EV isolation and characterisation methods, well‑defined clinical endpoints and regulatory alignment. Integrating EV research with longitudinal sampling and multicentre study designs will be essential to determine whether EV‑based diagnostics or therapeutics can meaningfully inform disease prediction, patient stratification or intervention in T1D. As such, EVs represent a compelling avenue for continued investigation, but one that will require coordinated methodological and clinical advancement before routine clinical implementation can be realised.

### 8.1 A comparative analysis between EVs and other standard measures as a biomarker for clinical utility

Early predictive research established the core framework upon which modern T1D surveillance is built. The seminal longitudinal study by Ziegler et al.^78^ demonstrated that the presence of two or more islet autoantibodies confers an almost inevitable risk of progression to clinical T1D. This finding reframed autoantibody positivity from a correlational marker to a deterministic predictor of disease. Subsequent work by Xu et al.^79^ and Sosenko et al.^80^ advanced this framework by integrating immunologic markers with metabolic indices, particularly C‑peptide decline. These efforts culminated in highly accurate composite algorithms, including the Diabetes Prevention Trial–Type 1 Risk Score (DPTRS), capable of predicting short‑term disease progression in at‑risk individuals. Further studies illustrated how these foundational markers were operationalised within the TrialNet consortium to define disease stages and evaluate preventive interventions over decades.^81^ Collectively, these studies transformed T1D from a binary diagnostic event into a measurable, staged autoimmune process with quantifiable risk trajectories.

However, despite their clinical utility, these established markers primarily function as diagnostic and late‑stage prognostic tools, reflecting immune activation and metabolic dysfunction after substantial β‑cell damage has already occurred. Although population‑level risk prediction is robust, precise individualised timing of disease onset remains imprecise, highlighting the need for biomarkers that capture earlier biology and dynamic disease activity.

Recent efforts have sought to push T1D prediction further upstream by identifying pre‐seroconversion molecular signatures. Nakayasu and colleagues^82^ reported plasma proteomic changes particularly in complement and inflammatory pathways, detectable up to 6 months before autoantibody appearance, challenging the assumption that autoantibodies represent the earliest measurable sign of disease. Expanding on this concept, a recent generative artificial intelligence‐based framework integrating 50 circulating miRNAs produced a dynamic risk score that accurately predicted β‑cell loss and future insulin dependence across four multi‑ethnic cohorts (AUC = .84).^83^ These approaches redefine risk prediction as a systems‑level, adaptive process rather than a static threshold and exemplify prognostic biomarkers aimed at forecasting disease trajectory prior to overt immunologic or metabolic changes. Nonetheless, reliance on high‑throughput sequencing and advanced computational pipelines currently limits near‑term clinical translation.

The microbiome has emerged as an attractive non‐invasive biomarker source. A recent study demonstrated that salivary microbiome analysis outperformed dental plaque in distinguishing paediatric T1D from healthy controls in Kuwait, achieving an AUC of .94.^84^ In contrast, analysis of the TEDDY cohort revealed marked variability in microbiome‑based predictive performance depending on analytical choices, with AUCs ranging from non‑informative to moderately predictive.^85^ While microbiome‑based biomarkers show promise as diagnostic screening tools, their strong susceptibility to confounders such as diet, inflammation, BMI and transit time raises concerns regarding reproducibility and global generalisability.

As predictive models mature, clinical focus has increasingly shifted towards monitoring biomarkers that support actionable stratification, therapeutic decision‑making and longitudinal assessment. In studies of heterogeneous responses to the immunotherapy teplizumab, a 26‑gene blood transcriptomic signature differentiated responders from non‑responders, with T‑cell‐enriched profiles predicting therapeutic success.^86^ Complementing this mechanistic approach, validation of the urinary C‑peptide creatinine ratio (UCPCR) as a non‑invasive, room‑temperature stable assay achieved 100% sensitivity for distinguishing T1D from T2D in adults, with substantial cost reduction.^87^ These tools exemplify biomarkers optimised for repeated measurement and real‑world implementation, although transcriptomic signatures remain technically complex and UCPCR specificity may be reduced in advanced disease states.

Within this evolving landscape, EVs occupy a distinct intermediate position between established clinical markers and emerging omics‑based approaches. EVs have the potential to function across diagnostic, prognostic and monitoring contexts, depending on their biological origin and analytical resolution. Unlike autoantibodies and metabolic markers, EVs may capture early, cell‑resolved stress and immune signalling events originating from β‑cells or immune populations before overt autoimmunity or dysglycaemia, supporting diagnostic and early prognostic applications. Compared with circulating miRNA or bulk proteomic signatures, EVs offer greater biological specificity by retaining information about the state and origin of the parent cell. In longitudinal settings, EVs may additionally serve as sensitive monitoring biomarkers, reflecting dynamic changes in immune activity or β‑cell stress during disease progression or in response to immunomodulatory therapy. However, EV‑based approaches currently lag in translational maturity, with challenges including assay standardisation, scalability and the lack of large longitudinal validation cohorts. Accordingly, EVs should be viewed not as replacements for established biomarkers, but as complementary intermediates capable of bridging molecular discovery and clinical application when integrated with existing immune and metabolic measures.

### 8.2 Comparative evaluation of T1D biomarker platforms

Within this evolving landscape, biomarker platforms can be compared across three dimensions; (1) biological resolution, (2) clinical practicality and (3) translational maturity. Table 2 provides a comparative overview of major T1D biomarker platforms across these dimensions, highlighting their relative strengths, limitations and current stage of clinical development.

**TABLE 2:** Comparative overview of biomarker platforms for clinical utility in T1D.

Biomarker platform	Information captured	Sensitivity to early disease	Invasiveness	Scalability	Translational maturity	Key limitations
Islet autoantibodies/C peptide	Adaptive autoimmunity, β cell function	Moderate–late	Invasive	High/low–moderate	High (clinically established)	Reflect established immune damage; limited timing precision
Circulating miRNAs/proteomics	Systemic immune and stress responses	High (pre‐seroconversion possible)	Invasive	Moderate–low/high	Moderate (early validation)	Requires high throughput sequencing and advanced analytics
Microbiome profiles	Host–microbe interactions	Variable	Non‐invasive (stool/saliva)	High/moderate	Low–moderate	Strongly confounded by diet, geography and analytical choices
Extracellular vesicles (blood/urine)	Cell type‐specific stress, immune signalling, tissue origin	High (cell resolved, dynamic)	Minimally invasive	Moderate	Low–moderate (pilot stage)	Assay heterogeneity; limited longitudinal validation

As summarised in Table 2, EVs occupy a distinct intermediate position between established clinical markers and emerging omics‑based approaches. Unlike autoantibodies or metabolic markers, EVs have the potential to capture early, cell‑resolved stress and immune signalling events, including signals originating from β‑cells and immune populations prior to overt autoimmunity. Compared with circulating miRNA or bulk proteomic signatures, EVs offer greater biological specificity, as their cargo reflects the state and origin of the parent cell. Relative to microbiome‑based biomarkers, EVs are less susceptible to environmental confounding and can be sampled repeatedly from standard clinical biofluids such as blood or urine. However, EV‑based approaches currently lag in translational maturity, with challenges including assay standardisation, scalability and the absence of large longitudinal validation cohorts. Accordingly, EVs should be viewed not as replacements for established biomarkers, but as complementary intermediates with the potential to bridge molecular discovery and clinical application when integrated with existing immune and metabolic measures. Additionally, to enhance the interpretability, reproducibility and cross‑study comparability of EV biomarker studies in T1D, we propose a T1D‑specific minimal reporting checklist to be reported alongside existing MISEV guidelines. While MISEV establishes essential standards for EV isolation, characterisation and analytical rigor, it does not capture disease‑specific clinical, immunological and metabolic variables that are fundamental for contextualising EV signals within the heterogeneous pathophysiology of T1D.

At a minimum, studies should report islet autoantibody status including number and specificity, T1D stage at the time of sampling and disease duration or time since seroconversion or diagnosis. Glycaemic status, as reflected by HbA1c, and residual β‑cell function, assessed by fasting or stimulated C‑peptide, should be reported with assay methodology clearly specified. The use of concurrent immunomodulatory or metabolic therapies at the time of sampling should also be detailed. Finally, EV‑specific methodological parameters including the isolation approach, purity and characterisation metrics, and whether EVs were analysed in bulk or at the single‑vesicle level should be explicitly reported, alongside study design features such as longitudinal sampling and clinical setting. These should be systematically documented to account for confounding influences on EV composition and abundance. Where feasible, details of longitudinal sampling design, clinical setting and concurrent immunomodulatory or metabolic therapies should also be reported.

Systematic inclusion of these core T1D‑specific variables, together with detailed comorbidity data and the judicious use of disease‑ or cell‑type‑specific EV enrichment strategies when possible, will enhance biological specificity, reduce misattribution of EV signals arising from disease heterogeneity and accelerate the translation of EV‑based biomarkers into clinically meaningful tools for T1D staging, disease monitoring and assessment of therapeutic response.

## 9 BOTTLENECK IN UTILISING EV AS A T1D BIOMARKER

**FIGURE 2:**
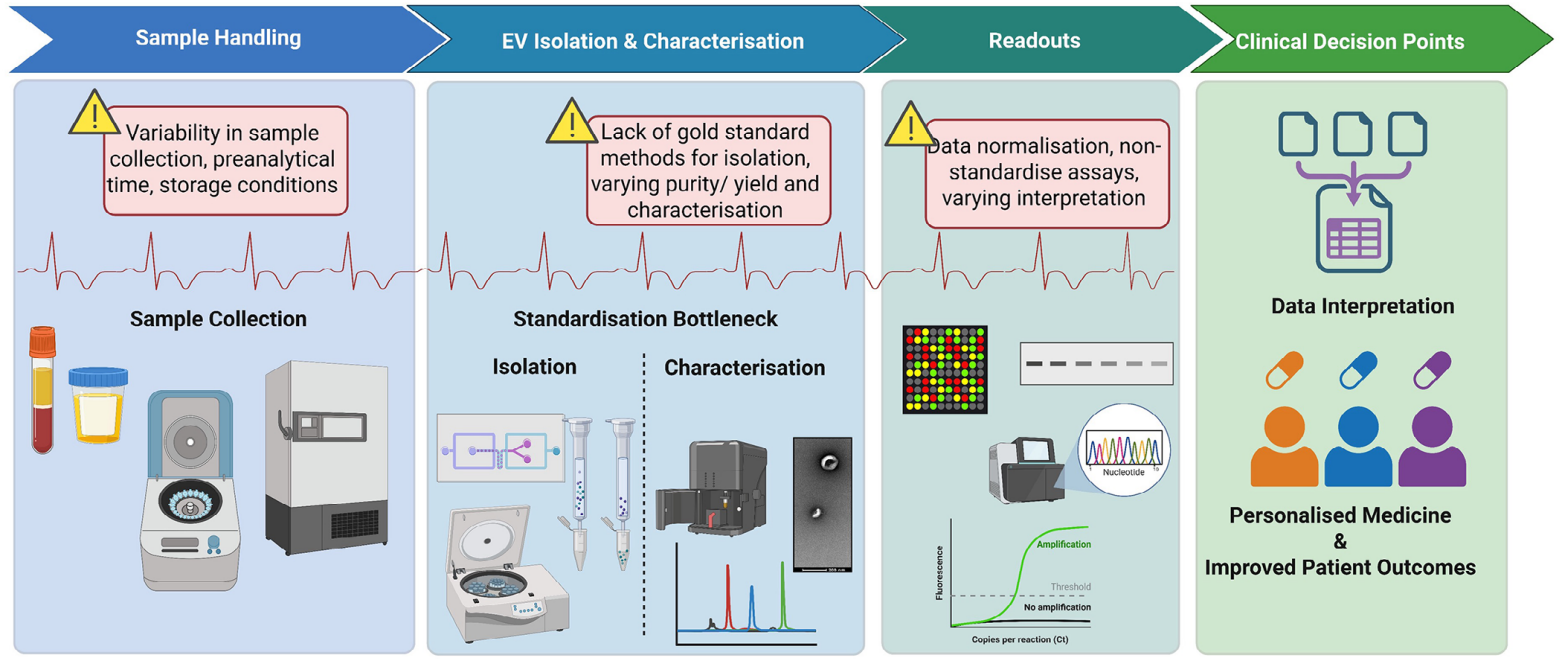
Technical and analytical bottlenecks across the EVs workflow.

Although most evidence remains preclinical, emerging clinical studies and early‑phase trials underscore the translational potential of EV‑based strategies to shift T1D care from reactive treatment towards proactive, personalised intervention. To fully realise this promise, several key priorities must be addressed in future research. These include the standardisation of EV isolation (Table 3), characterisation and reporting frameworks (Figure 2) to ensure reproducibility and cross‑study comparability; the validation of EV‑associated protein, RNA, lipid and DNA biomarkers in large‑scale, longitudinal cohorts that capture diverse genetic, environmental and developmental contexts and the integration of EV‑derived signatures with existing clinical, immunological and metabolic risk models to improve disease stratification and prediction of progression.

**TABLE 3:** Summary of studies on extracellular vesicles in type 1 diabetes with various isolation methods.

Study	Source of EVs	Isolation method	Study type	Key findings
^125^	Serum	Total isolation kit, ExoQuick, ultrafiltration and ultracentrifugation (UC)	Methodological	This study compared different purification methods and emphasised that ultrafiltration yielded better size distribution and purity of EVs compared with others.
^35^	Human pancreatic islets from T1D donors	UC	Clinical + in vitro	Observed that beta cell‐derived EVs carried miR‐21‐5p, which was increased in response to inflammatory cytokines, acting as a potential biomarker for T1D.
^51^	MIN6 β‐cells	Density gradient UC	In vitro	The paper reported that stressed β‐cell EVs carried autoantigens such as insulin, GAD65 and IA‐2 and the EVs may act as messenger that recruit and activate immune cells, potentially driving autoimmune destruction of insulin‐producing cells.
^126^	Plasma	Size‐exclusion chromatography (SEC)	In vitro	This study reported that the DOC2B, a vesicle priming protein essential for glucose‐stimulated insulin secretion, is actively sorted into EVs released by β‐cells and could serve as biomarkers for β‐cell health and autoimmune activity in diabetes.
^127^	Circulating EVs from T1D patients	UC, density‐based UC, modified ExoQuick and SEC	In vitro	The paper demonstrates that circulating EVs from T1D patients are pro‐inflammatory and directly contribute to β‐cell damage. These EVs carry immune‐activating molecules such as TNF, CXC10, miR21 and miR155 which can induce cytotoxicity in pancreatic β‐cells and amplify autoimmune pathways, thereby playing a pathogenic role in T1D progression.
^42^	Islets of Langerhans derived	UC	In vitro	This paper reported that EVs released from human islets of Langerhans can activate peripheral blood mononuclear cells (PBMCs), providing evidence that EVs act as immune messengers in T1D.
^128^	Plasma	UC	Clinical	This paper reported on a panel of dysregulated EV miRNAs in plasma from patients with long‐duration T1D. The most notable ones included miR‐21‐5p, miR‐24‐3p, miR‐126‐3p, miR‐146a‐5p, miR‐150‐5p, miR‐181a‐5p, miR‐210‐3p and miR‐342‐3p, highlighting their potential as biomarkers for disease monitoring.
^129^	Adipose tissue‐derived MSC	UC	In vivo	This study reported that the adipose tissue‐derived MSC exosomes achieved similar immunomodulatory results to stem cell transplantation. By increasing the Treg population, the exosomes effectively ‘re‐educated’ the immune system to stop attacking the pancreatic β‐cells, providing a potential safer alternative to live cell injections in treating T1D.
^77^	Human bone marrow‐derived MSC	UC	In vitro with exosomes isolated from clinical samples	This paper reported that MSCs and MSC‐derived Evs induces a regulatory, immature dendritic cells profile marked by enhanced IL‐10 production and reduced IFN‐γ responses. This can contribute to inhibition of T cell responses to islet antigens.
^130^	Plasma	SEC + immunoaffinity	Clinical	In this longitudinal study, researchers found a drop if the donor‐islet exosomes were detected on Day 1001, which was 97 days before the patient became hyperglycaemic. This study showed the circulating islet EVs has the potential to be a diagnostic tool for recurrent autoimmune T1DM after islet transplantation as the exosomes cargo showed presence of GAD65 autoantigen, proposing exosomes as a methods of autoantigen trafficking and presentation.

Sex and age are increasingly recognised as important biological modifiers of EV abundance, composition and functional signalling. Sex‑specific differences in EV profiles including concentration and cargo have been documented between males and females, with sex hormones acting as key upstream regulators of EV biogenesis and cargo sorting.^88^, ^89^, ^90^ Both androgen^91^, ^92^, ^93^ and oestrogen signalling^94^ has been shown to influence EV release and nucleic acid loading suggesting direct hormonal control of EV dynamics. These differences are particularly relevant to autoimmune diseases, including T1D, in which sex‑biased immune responses and disease trajectories are well described.^10^ Ageing is associated with EV signatures enriched for inflammatory, senescence‑associated and metabolic stress pathways, and^95^, ^96^ with increased contributions from immune‑ and endothelial‑derived EVs,^97^, ^98^ potentially obscuring low‑abundance tissue‑specific EV populations. These changes are particularly relevant in T1D, which spans both paediatric‑ and adult‑onset disease and exhibits marked heterogeneity in immune‑mediated β‑cell destruction. Sex and age effects on EV biology are not independent but interact dynamically where puberty represents a critical inflection point during which hormonal transitions reshape immune function and EV release patterns, while age‑dependent accumulation of comorbidities further influences EV composition and cellular origin.

Importantly, many methodological bottlenecks arise even earlier in the EV workflow, at the level of pre‑analytical sample handling. Pre‑analytical handling is a critical determinant of EV integrity, yield and cargo composition, and represents a major source of variability in EV‑based biomarker studies. To preserve EV stability, biofluid collection should use standardised tubes and protocols, with particular attention to minimising haemolysis and platelet activation for plasma samples, as ex vivo platelet and erythrocyte activation can substantially distort EV abundance and composition.^99^ Delays between sample collection and processing should be kept as short as possible, with multiple studies and consensus guidelines recommending processing within 2–4 h at 4°C to limit EV degradation and artefactual vesicle release.^100^ Where immediate processing is not feasible, samples should be centrifuged to remove cells and debris prior to storage to prevent freeze–thaw‑induced release of non‑vesicular particles.^101^ EV‐containing plasma or urine is best stored at −80°C, as repeated freeze–thaw cycles have been shown to alter EV concentration, size distribution, membrane integrity and RNA and protein cargo.^102^ Therefore, aliquoting samples before freezing is strongly recommended. The use of protease and RNase inhibitors may further protect EV cargo, particularly for downstream proteomic or transcriptomic analyses, especially in urine‐derived EV preparations.^100^ Importantly, current MISEV guidelines emphasise explicit reporting of key pre‐analytical variables including collection tubes, processing time, centrifugation steps, storage temperature and duration and number of freeze–thaw cycles to enable reproducibility and meaningful cross‐study comparison.^16^ In a nutshell, careful optimisation and transparent reporting of pre‐analytical conditions are especially critical in paediatric and longitudinal T1D studies, where limited sample volumes and repeated sampling heighten sensitivity to handling‐related artefacts and bias downstream EV analyse.

Another major challenge in utilising circulating EVs as biomarkers is the substantial background noise arising from EV heterogeneity. EVs are released by virtually all cell types, and those derived from diseased cells coexist with a vast excess of EVs originating from healthy tissues, making the detection of disease specific signals particularly challenging.^103^

The overall concentration of circulating EVs in plasma in a healthy individual is estimated to be approximately 10^10^–10^1^ ^3^ particles/mL.^104^ The majority of plasma EVs originate from haematopoietic cells, whereas EVs derived from peripheral tissues collectively account for only approximately .2% of the circulating EV pool. Among tissue‐derived EVs, adipose tissue represents the dominant contributor, while pancreatic EVs constitute less than 1% of total circulating EVs.^105^ Notably, this proportion is known to increase in T1D.^22^, ^23^, ^24^ The low baseline abundance of pancreatic EVs presents a significant obstacle for their reliable detection and isolation. Another significant obstacle to the clinical translation of EV in autoimmune diseases such as T1D is systemic inflammation, which induces a massive release of EVs from immune cells. This creates a ‘loud’ background of redundant inflammatory signals that obscures the comparatively ‘quiet’ signal of disease‐specific EVs. Another important practical limitation for EV biomarker studies especially in paediatric T1D populations is the requirement for relatively large sample volumes by many conventional EV isolation and characterisation methods. Plasma‐based EV workflows often necessitate large volume inputs to achieve adequate yield and purity,^106^ which may be infeasible or ethically challenging in young children, particularly in longitudinal or early‑stage disease studies. These constraints can limit repeated sampling, reduce study feasibility and introduce selection bias towards older or less clinically vulnerable participants.

To address these challenges, advanced isolation strategies such as immunoaffinity‐based purification using target specific ligands conjugated to magnetic beads, as well as microfluidic technologies, have been developed to enrich EV populations and reduce contamination from proteins and lipoproteins.^107^, ^108^, ^109^, ^110^ Furthermore, the application of quantum dot‐based immunolabelling can enhance detection sensitivity and minimise background interference in analytical techniques such as nanoparticle tracking analysis.^111^, ^112^, ^113^ Accordingly, paediatric EV studies should prioritise low‑input and high‑sensitivity methodologies, such as microfluidic isolation platforms,^108^, ^109^ immunoaffinity‑based enrichment strategies^110^ and single‑vesicle analytical approaches,^114^ which are compatible with small sample volumes. Explicit reporting of input volume requirements and optimisation for paediatric sampling will be essential to ensure equitable inclusion of young children and to support the clinical translation of EV‑based biomarkers in early‑onset T1D.

Key sources of variability arise during sample handling (collection, pre‑analytical timing and storage), EV isolation and characterisation (lack of gold‑standard methods affecting purity, yield and profiling) and downstream readouts (data normalisation and heterogeneous analytical pipelines). These cumulative limitations influence data interpretation and clinical decision‑making, highlighting the need for improved standardisation to enable robust, reproducible and translatable EV research.

Beyond bulk EV profiling, single‐EV sequencing approaches offer important advantages by preventing non‐specific inflammatory RNA derived from immune EVs from being conflated with autoantigen‐encoding RNA originating from disease‐relevant tissues.^115^

While the EV surface may be dominated by common inflammatory markers, the EV cargo contains highly specific autoantigens capable of definitively identifying tissue or disease origin.^29^ Thus, integration of selective isolation strategies with high‑resolution EV analysis therefore enables more precise elucidation of the cellular origin and functional relevance of disease‑associated EV subpopulations.

The interpretation of EV‑based readouts is further complicated by common comorbidities in T1D. Autoimmune thyroid disease^116^, ^117^ and celiac disease,^118^, ^119^ both highly prevalent in individuals with T1D, are associated with chronic immune activation and tissue‐specific inflammation that can independently reshape circulating EV composition. Similarly, overweight and obesity are increasingly observed in paediatric and adult T1D populations^120^ and are known to influence EV release from adipose tissue, immune cells and endothelium,^121^ introducing metabolic and inflammatory EV signatures that overlap with islet stress and dysfunction.^122^ To mitigate these confounding effects, systematic collection of detailed comorbidity and metabolic phenotyping data – including autoimmune status, body mass index and markers of systemic inflammation – is essential. Where feasible, disease‑ or cell‑type‑specific EV enrichment strategies, such as immunocapture of islet‑, adipose‑ or immune‑derived EVs, may further improve specificity.

Age‑related variation in EV profiles represents an additional source of biological heterogeneity. EV concentration, size distribution and cargo composition change across the life course, reflecting immune maturation in early life and immunosenescence and tissue ageing in adulthood

Finally, the robust interpretation of EV‐based biomarker in T1D requires the inclusion of a minimal but principled panel of positive and negative controls that address both EV identity and disease relevance. At a minimum, studies should include canonical EV markers such as CD9, CD63, CD81, TSG101 or ALIX^16^ as positive controls to confirm successful isolation of bona fide EVs, alongside immune‑derived EV markers such as CD45, CD3, CD14, HLA class I/II^123^, ^124^ that are expected to be enriched in the inflammatory milieu characteristic of T1D and provide contextual information on immune background noise. Disease‑specific positive controls should include low‑abundance but highly informative β‑cell or islet‑associated markers such as INS, IAPP, G6PC2, PCSK1, MAFA or miR‑375 at the protein or RNA level to anchor biological relevance to β‑cell stress or loss. Negative controls are equally essential and should encompass markers of non‑EV contaminants such as albumin, apolipoproteins, fibrinogen or ER proteins to assess sample purity, as well as disease‑irrelevant tissue markers to confirm tissue specificity. Inclusion of autoantibody‑negative, normoglycaemic, age‑ and sex‑matched healthy controls is critical to define baseline EV variability, particularly in early‑stage T1D. Together, this minimal control framework strengthens biological specificity, mitigates confounding from immune and plasma‑derived EV, and enhances reproducibility and cross‑study comparability in T1D‑EV biomarker research.

## 10 CONCLUSION AND FUTURE DIRECTIONS

**FIGURE 3:**
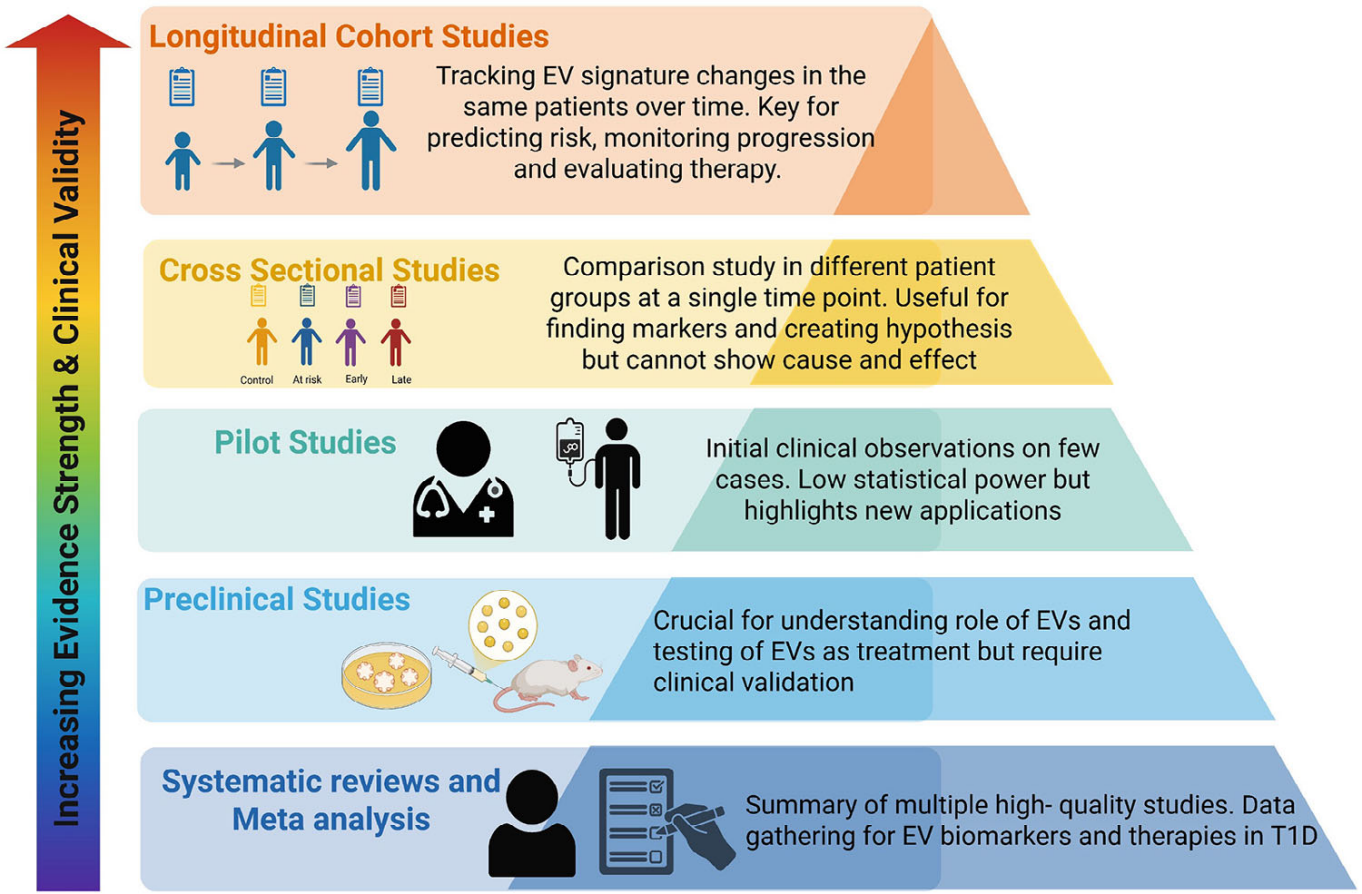
A hierarchical framework of study designs inEVs research for T1D.

EVs represent a compelling opportunity to advance the diagnosis and management of T1D. Their molecular signatures position EVs as highly informative, non‐invasive biomarkers for early disease detection, disease stratification and longitudinal monitoring. In parallel, the immunomodulatory and regenerative properties of EVs underscore their promise as therapeutic agents capable of modulating autoimmune responses and preserving residual β cell function. Overcoming the technical, biological and translational challenges outlined above will be essential to enable clinical implementation of EV‐based diagnostics and therapeutics. As summarised in Figure 3, the evidential framework supporting EV translation strengthens progressively from preclinical and pilot investigations to cross sectional and, ultimately, longitudinal cohort studies, which are crucial for establishing clinical validity and utility. Looking ahead, the integration of EV technologies into precision medicine approaches has the potential to facilitate earlier intervention, preserve β cell mass and mitigate long‐term complications, thereby marking a paradigm shift in the prevention and management of T1D.

The hierarchy of study designs underpinning EV research in T1D, organised by increasing evidence strength and clinical validity. This highlights the increasing evidence strength from preclinical and pilot studies through cross‐sectional and longitudinal cohort studies, culminating in systematic reviews and meta‐analyses. The framework emphasises how different study types contribute uniquely to understanding EV biology and advancing their clinical translation.

## AUTHOR CONTRIBUTIONS

Nanthini Jayabalan and Carlos Salomon drafted the original manuscript. Kriti Joshi and Tony Huynh contributed to the clinical conceptualisation of the review, while Flavio Carrion provided expertise on the immunological aspects. All authors contributed to data interpretation, critically reviewed the manuscript and approved the final version for publication.

## FUNDING INFORMATION

The authors have nothing to report.

## CONFLICT OF INTEREST STATEMENT

The authors declare no conflicts of interest.

## ETHICS STATEMENT

The authors have nothing to report.

## CONSENT

The authors have nothing to report.

## AI USE DECLARATION

AI tools were used only for English grammar correction and language polishing.

## Data Availability

The authors have nothing to report.
